# Identifying depression’s genetic role as a precursor to sepsis and increased mortality risk: Comprehensive insights from mendelian randomization analysis

**DOI:** 10.1371/journal.pone.0300275

**Published:** 2024-05-28

**Authors:** Qingyi Zhou, Qili Shen, Xiaohua Chen, Lichun Yang, Qiang Ma, Liang Chu

**Affiliations:** 1 Second Affiliated Hospital of Bengbu Medical College, Bengbu, China; 2 First Affiliated Hospital of Bengbu Medical College, Bengbu, China; NMC Royal Hospital / The National Research Centre, UNITED ARAB EMIRATES

## Abstract

**Background:**

Previous retrospective studies have shown a correlation between depression and increased risk of infections, including a moderate rise in sepsis likelihood associated with severe depression and anxiety. To investigate the potential causal links between depression, sepsis, and mortality risks, while considering confounding factors, we employed a Mendelian randomization (MR) approach.

**Methods:**

In this two-sample Mendelian randomization study, we analyzed data from a large-scale genome-wide association study on depression, involving 807,553 European individuals (246,363 cases, 561,190 controls). We extracted SNP associations with sepsis and 28-day mortality from UK Biobank GWAS outcomes. The correlation analysis primarily employed the inverse-variance weighted method, supplemented by sensitivity analyses for heterogeneity and pleiotropy assessment.

**Results:**

Our analysis revealed a potential causal link between depression and an increased risk of sepsis (OR = 1.246, 95% CI: 1.076–1.442, *P* = 0.003), but no causal association was found with sepsis-induced mortality risk (OR = 1.274, 95% CI: 0.891–1.823, *P* = 0.184). Sensitivity analyses confirmed the robustness of these findings.

**Conclusions:**

We identified a potential causal association between depression and heightened sepsis risk, while no link was found with sepsis-induced mortality. These findings suggest that effective management of depression could be important in preventing sepsis.

## Introduction

Sepsis, characterized by a dysregulated host response to infection leading to organ dysfunction, remains a significant global public health issue with devastating consequences [1]. It imposes a substantial burden of morbidity and mortality worldwide, claiming the lives of approximately 3 to 4 million individuals each year [2]. This life-threatening condition sets off a complex and potent inflammatory cascade within the body, resulting in the dysfunction of multiple organ systems [3–5]. In severe cases, sepsis can progress to multiple organ failure, further exacerbating the risk to patients’ lives. The epidemiological inquiries conducted to date have unequivocally identified sepsis as a paramount driver of mortality and a critical public health concern on a global scale. Recognizing its impact, efforts to raise awareness, and identify potential risk factors are of utmost importance [6, 7].

Depression, an all-encompassing mood disorder, stands as a prevalent mental illness with far-reaching implications for individuals and society as a whole [8]. Its definition is derived from meticulous clinical symptomatology and precise diagnostic criteria, encompassing enduring experiences of profound sadness, pervasive feelings of helplessness, and a notable loss of interest or pleasure in once-enjoyable activities, known as anhedonia. From an epidemiological perspective, depression assumes a paramount position as a formidable global health challenge, exacting a substantial toll upon individuals and communities alike [9–12]. Building on existing evidence, it appears that depression alone may contribute to an elevated risk of developing infections. Notably, severe symptoms of depression and anxiety have been correlated with a moderately heightened risk of sepsis [13–16]. However, the limitations of traditional observational studies, particularly concerning confounding biases and reverse causality, hinder definitive causal inferences. Associations between depression, sepsis risk, and mortality may be confounded by factors like comorbidities, BMI, and lifestyle [15]. The paucity of evidence from randomized controlled trials (RCTs) further challenges the establishment of clear causality, leaving the relationship between depression, sepsis risk, and mortality not fully established.

The Mendelian randomization (MR) approach represents a popular methodology for assessing causal inference between exposure and outcome. Notably, MR leverages single nucleotide polymorphisms (SNPs) as instrumental variables (IVs), thereby constituting a quasi-RCT, as the genetic variants are ascribed randomly at the time of conception and are not influenced by confounding factors. Therefore, the aim of this study was to undertake the MR approach to scrutinize the potential causalities between depression and the risk of sepsis and sepsis-related mortality.

## Methods

### Study design

**Fig 1:**
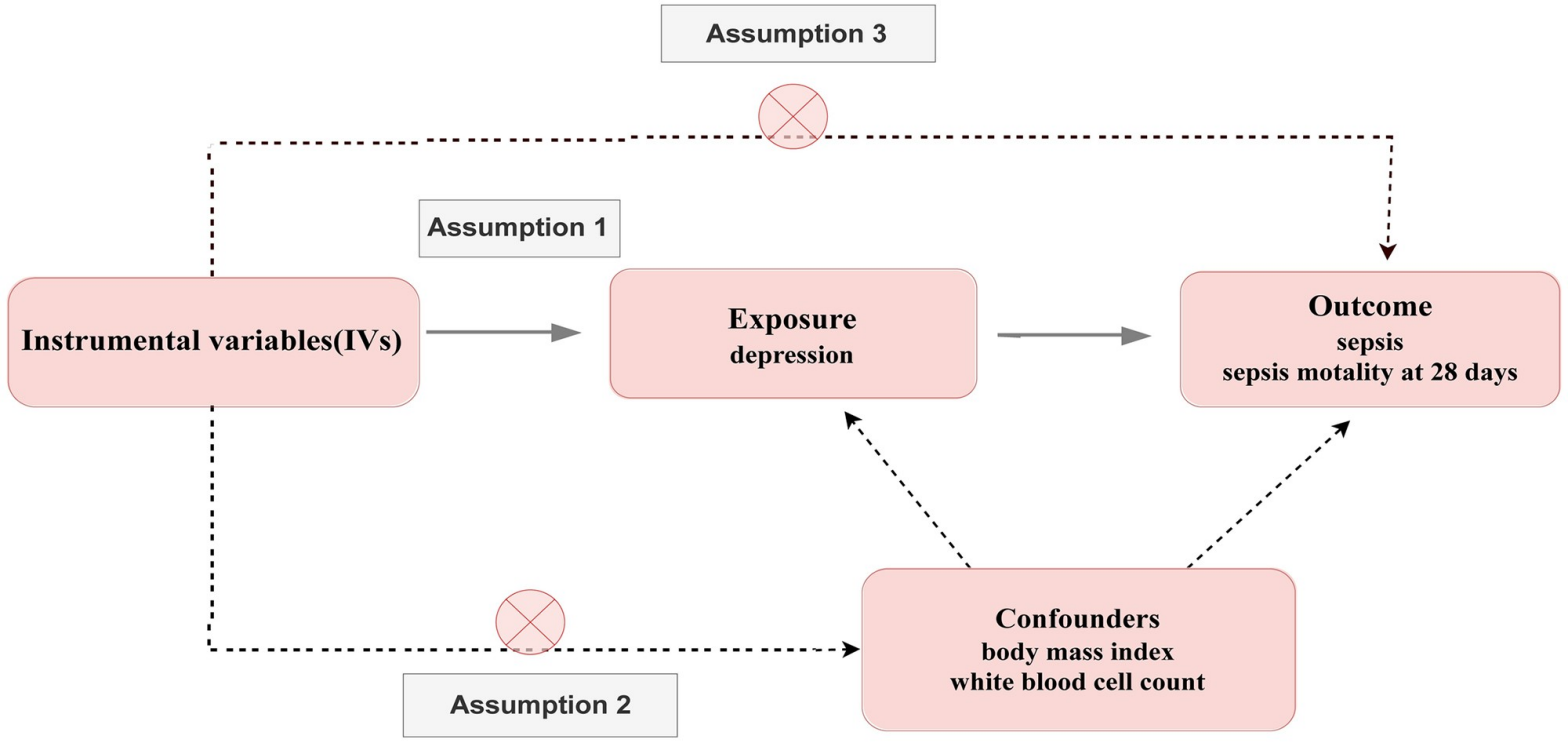
Overall design of the present study. The MR analysis was based on three assumptions.

An overview of the MR study design is shown in Fig 1 The MR analysis was based on three assumptions [17, 18]. (1) The selected IVs is closely related to the exposure. (2) The selected genetic IVs (Instrumental variables) are devoid of known confounding factors. (3) The selected single-nucleotide polymorphisms (SNPs) had no direct influence on the outcome but only influenced outcome indirectly through the exposure. The MR study outcomes included primary and secondary outcomes; the primary outcome was sepsis and the secondary outcomes were sepsis-induced mortality.

### Date sources

A large GWAS meta-analysis of depression involved 807,553 individuals of European ancestry (246,363 cases and 561,190 controls) from the three largest GWASs [19]. Depression was defined as a debilitating psychiatric illness that was typically associated with low mood and anhedonia. We obtained genetic association estimations for SNPs linked to sepsis and sepsis mortality at 28 days from the GWAS outcomes of the UK Biobank [20, 21]. Specifically, sepsis cases were identified using International Classification of Diseases (ICD) 10th edition codes A02, A39, A40, and A41, which are consistent with the definitions used in recent literature. These estimations were easily accessed via the Integrative Epidemiologic Unit (IEU) GWAS database at https://gwas.mrcieu.ac.uk/. Table 1 summarizes the GWAS data used in this study.

**Table 1:** Summary information of the data sets.

Traits	Data sources	Year	Samplesize (cases/controls)	Ancestry	Sex
Depression	A meta-analysis from Howard DM	2019	807553(246363/561190)	European	Males and Females
Sepsis	UK Biobank	2021	486484(11643/474842)	European	Males and Females
Sepsis mortality at 28 days	UK Biobank	2021	486484(1896/484588)	European	Males and Females

A meta-analysis from Howard DM, a large-scale genome-wide association study on depression.

### Selection of instrumental variables

In our investigation into the genetic underpinnings of depression, we identified 102 SNPs exhibiting a robust association with the disorder, surpassing the Genome-Wide Association Studies (GWAS) significance threshold (P<5e-8). This association remained after adjusting for sex and age variables, as detailed in supporting information files. These SNPs are implicated in gene pathways critical to synaptic structure and neurotransmission.

Recognizing the potential bias from linkage disequilibrium (LD) in Mendelian Randomization (MR) studies, we conducted a clumping procedure (R^2^ = 0.001, kb = 10000) using the European cohort from the 1000 Genomes Project. This process involved removing SNPs with higher P values in instances where r^2^ >0.001, as well as discarding SNPs absent from the LD reference panel. Additionally, any SNPs that were ambiguous or palindromic, exhibiting non-concordant alleles or ambiguous strands, were excluded. All SNPs obtained were strong IVs, and F values (range 147.8–374.5) were all greater than the recommended threshold of 10 (S1 Table), indicating that there was no bias caused by weak IVs in the study [22]. Online tools (https://shiny.cnsgenomics.com/mRnd/) were used to calculate each SNPs’ MR analysis of statistical power of sepsis [23].

To satisfy assumption 1, the confounding factors such as body mass index, white blood cell count were identified in the datasets using the PhenoScanner database (http://www.phenoscanner.medschl.cam.ac.uk/). A total of 4 SNPs associated confounding factors (rs1095626, rs200949, rs2568958, rs3823624) were excluded. The remaining SNPs were included in the subsequent analysis.

### Statistical analysis and sensitivity analysis

In our study, we applied multiple methods including Inverse Variance Weighted (IVW), Weighted Median Estimator (WME), MR-Egger, weighted, and simple models to assess the causal link between depression and sepsis risk. Notably, The IVW approach applies the reciprocal of outcome variance for weighting and overlooks the intercept in regression [24, 25]. In contrast to the IVW method, the MR-Egger approach accounts for an intercept and employs the inverse of the instrumental variables’ variance for fitting. Under the assumption of Instrument Strength Independent of Direct Effect, the findings from MR-Egger and MR-PRESSO remain valid, even in the presence of pleiotropic SNPs [25, 26]. The WME method orders SNP estimates by weight, effective when over 50% of SNPs are valid instruments. The weighted method assesses causality based on large SNP clusters. Due to its higher statistical efficacy, the IVW method was primarily used for identifying causality [18]. Results are presented as odds ratios (OR) and 95% confidence intervals (CI).

To ensure the reliability of our Mendelian Randomization (MR) results, we first utilized the MR-Egger regression intercept’s P-value to check for pleiotropic SNPs. This regression also helps identify research bias in meta-analyses. We then employed Cochran’s Q test for assessing the heterogeneity in effect size estimations of individual SNPs. Additionally, to confirm the correct causal direction between outcomes and exposure, we applied the Steiger directionality test. Further robustness was established by iteratively excluding each instrumental variable (IV), preventing undue influence from any single SNP. We visually represented IV’s directional horizontal pleiotropy using funnel plots, each based on a single Wald ratio for SNPs. Finally, the MR-PRESSO test was conducted to detect pleiotropy, removing outliers and re-evaluating the effect estimates for enhanced accuracy.

The occurrence of false positives was reduced using the Bonferroni correction (P = 0.05/2). All analyses were performed using the “TwoSampleMR” and “MR-PRESSO” packages of the RStudio application.

## Results

### Primary MR analysis: Influence of the depression on sepsis

**Fig 2:**
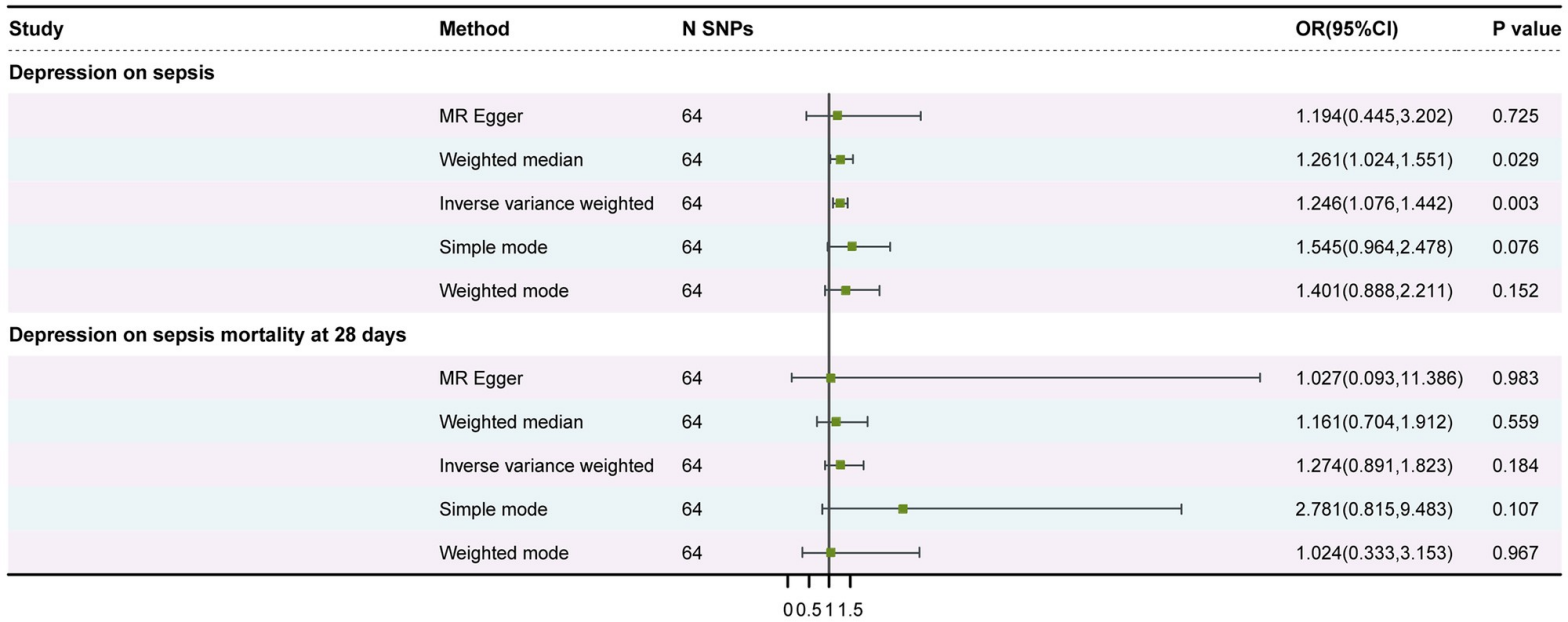
Mendelian randomization results of the causal effect of depression on the risk of sepsis and sepsis mortality. Forest plots of causality. (A) Primary outcome (sepsis); (B) Secondary outcome (Sepsis mortality at 28 days).

**Fig 3:**
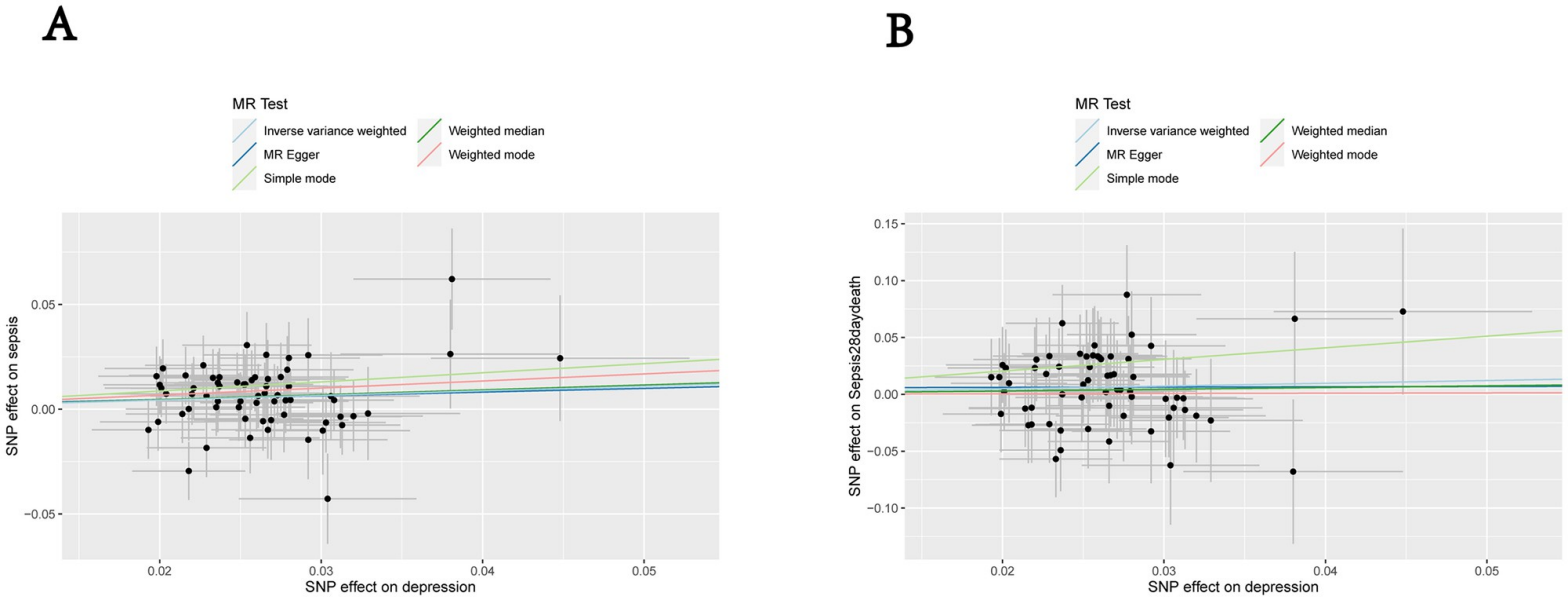
Scatter plots of causality. (A) Primary outcome (sepsis); (B) Secondary outcome (Sepsis mortality at 28 days).

**Fig 4:**
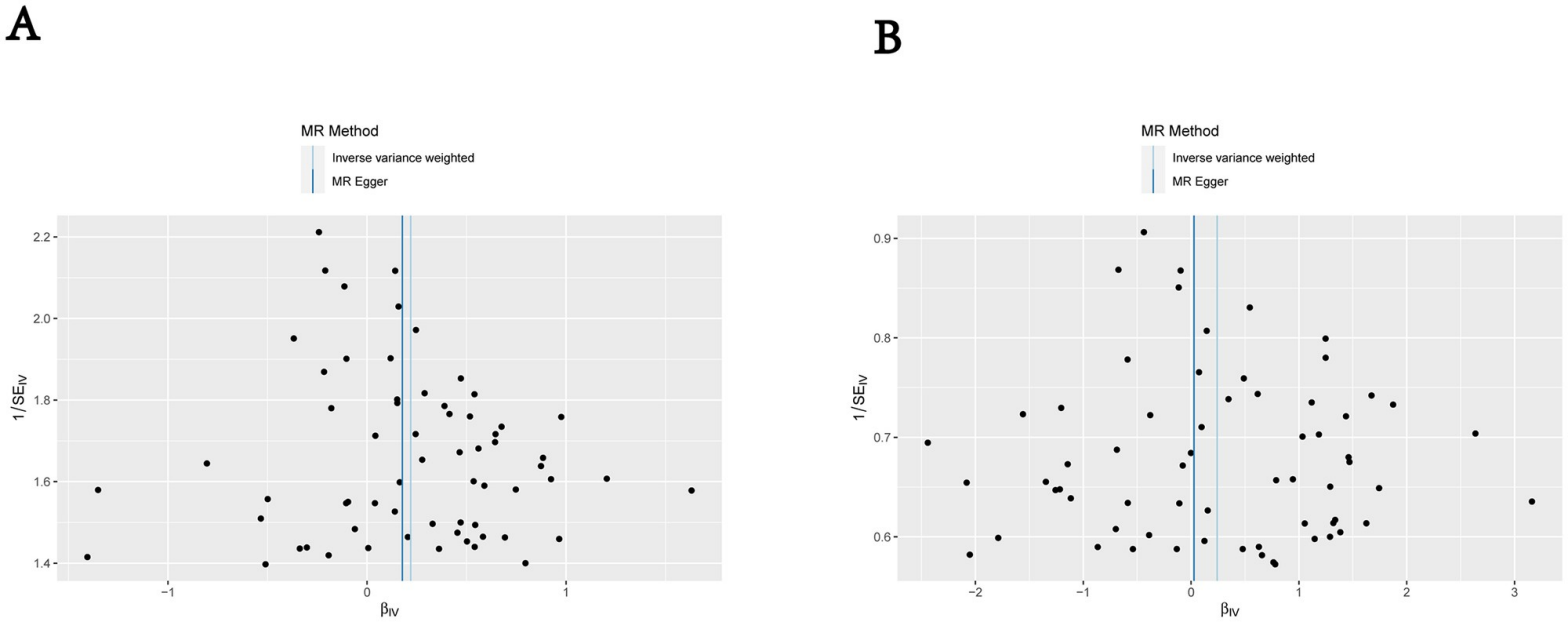
Funnel plots of causality. (A) Primary outcome (sepsis); (B) Secondary outcome (Sepsis mortality at 28 days).

Fig 2 displays the MR analyses. Our IVW-MR approach revealed that an increased depression was significantly associated with an increased risk of sepsis (OR = 1.246, 95% CI: 1.076–1.442, P = 0.003). The results were obtained using the WME analysis (OR = 1.261, 95% CI: 1.024–1.551, P = 0.029). There was no statistically significant difference in the MR-Egger analysis (OR = 1.194, 95% CI: 0.445–3.202, P = 0.725). Scatter plots and Funnel plots were showed in Figs 3 and 4. The removing a certain SNP using the leave-one-out method (S1 Fig). There was no significant heterogeneity in Cochran’s Q test (Q = 46.66015, P = 0.9387078). No outlier was identified by the MR-PRESSO Outlier Test. The MR pleiotropy test showed no horizontal pleiotropy by the MR-Egger regression (intercept = 0.001, se = 0.0129, P = 0.933) and MR-PRESSO Global Test (P = 0.993) (Table 2).

**Table 2:** Mendelian randomization results of the causal effect of depression on the risk of sepsis and sepsis mortality.

Outcome	Method	N	OR	95% LCI	95% UCI	P	Heterogeneity	Pleiotropy	MR-PRESSO
							P value	P value	Global test
Sepsis									0.925
	MR Egger	64	1.194	0.445	3.202	0.725	0.927	0.993	
	Weighted median	64	1.261	1.024	1.551	0.029			
	Inverse variance weighted	64	1.246	1.076	1.442	0.003	0.939		
	Simple mode	64	1.545	0.964	2.478	0.076			
	Weighted mode	64	1.401	0.888	2.211	0.152			
Sepsis mortality									0.993
	MR Egger	64	1.027	0.093	11.386	0.983	0.991	0.859	
	Weighted median	64	1.161	0.704	1.912	0.559			
	Inverse variance weighted	64	1.274	0.891	1.823	0.184	0.993		
	Simple mode	64	2.781	0.815	9.483	0.107			
	Weighted mode	64	1.024	0.333	3.153	0.967			

N, nummber of single nucleotide polymorphism; P, P-value.

### Secondary MR analysis: Influence of depression on sepsis-induced mortality

Fig 2 displays the MR analyses. Our IVW-MR approach revealed that increased depression was not significantly associated with an increased risk of sepsis-induced mortality (OR = 1.274, 95% CI: 0.891–1.823, *P* = 0.184). This result was also reflected in the WME analysis (OR = 1.161, 95% CI: 0.704–1.912, *P* = 0.559). The MR-Egger analysis showed no statistically significant difference (OR = 1.027, 95% CI: 0.093–11.386, *P* = 0.983). The removal of SNPs using the leave-one-out method had no significant influence on the results (S1 Fig). Significant heterogeneity was observed in Cochran’s *Q* test (*Q* = 38.873, *P* = 0.993). No outliers were identified by the MR-PRESSO test. Neither horizontal pleiotropy nor significant differences were identified by the MR-Egger regression (intercept = 0.006, SE = 0.031, *P* = 0.860) and MR-PRESSO Global Test (*P* = 0.925) (Table 2).

## Discussion

Our MR study reveals causal relationships between the depression and sepsis (OR = 1.246; 95% CI: 1.076–1.442, P = 0.003), This result was not observed in sepsis-related mortality (OR = 1.274, 95% CI: 0.891–1.823, P = 0.184).

Sepsis, an acute infectious disease, elicits a profound systemic inflammatory response, exerting a pervasive and intricate influence on the immune system. Depression, through various pathways, has been implicated in increasing the risk of sepsis by modulating immune system function [11, 12, 27–31]. Several potential mechanisms can be elucidated.

Firstly, individuals grappling with depression consistently exhibit disruptions in immune regulation, showcasing compromised functionalities across pivotal immune cell types—T cells, B cells, and natural killer cells [11, 30, 32, 33]. These nuanced alterations collectively erode the immune system’s inherent capacity to effectively respond to and counteract infectious agents, setting the stage for heightened vulnerability. Secondly, a compelling association emerges between depression and an augmented inflammatory response, underscored by elevated levels of inflammatory mediators, notably cytokines like interleukin-6 and tumor necrosis factor-alpha, in affected individuals [14, 29, 34–36]. This heightened inflammatory milieu not only signifies a potential risk but also hints at the propensity to induce overactivation or dysregulation of the immune system. This dysregulation, in turn, amplifies susceptibility to infections, including the formidable condition of sepsis. Thirdly, the narrative of depression unfolds in concert with an accentuated stress response, triggering the heightened release of adrenal corticosteroids [37, 38]. Prolonged exposure to stressors, a hallmark of depression, may exert a pervasive influence, compromising the functionality of the immune system and diminishing its efficacy in responding to infections. Moreover, depression extends its influence into the domain of lifestyle factors, where inadequate sleep, unhealthy dietary habits, and a lack of exercise emerge as significant players [39, 40]. These intricate interconnections between depression and lifestyle factors further compromise the functioning of the immune system, contributing substantively to an elevated risk of infections.

Previous retrospective studies have shown a correlation between depression and increased risk of infections. A investigation, leveraging data from the UK Biobank, has unearthed a potential link between depression and an elevated susceptibility to hospitalization due to infections [41]. The study, involving 976,398 participants and spanning from 1995 to 2012 in Denmark, revealed that individuals with a history of depression may face a heightened risk of severe infections [16]. This prospective, population-based research sheds light on the enduring impact of depression on infection vulnerability, surpassing a specific post-depression timeframe. Notably, the study hints at a potential dose-response relationship, suggesting that the severity of depression may correlate with an increased risk of infections. However, the findings emphasize the necessity for rigorous additional research to thoroughly elucidate and validate the intricate connection between depression and infection risk. Expanding on these insights, a subsequent cohort study conducted in Denmark from 2005 to 2013 delved deeper into the association between unipolar depression and the 30-day mortality rate in adults hospitalized for infections. The outcomes indicated a subtle elevation in the risk of mortality within 30 days for individuals with depression following a severe infection [42].

However, previous retrospective studies have not resulted in a consensus on whether the depression has a direct effect on sepsis and sepsis-induced mortality [15, 16, 34, 41, 42]. Sepsis is a complex process affected by many factors, such as body mass index, white blood cell count and so on. These may act as confounding factors in the retrospective analysis of sepsis risk. This study is the first to evaluate the causal relationship between the depression and sepsis at the genetic level. using two GWAS datasets related to sepsis and five different models to evaluate causality.

Based on the results, a strong causal relationship has been revealed between depression and sepsis, indentifying a heightened risk of sepsis occurrence among individuals with depression. However, notably, no substantial correlation has been detected between depression and the 28-day mortality rate in sepsis patients. Although the findings indicate an absence of a direct link between depression and mortality in the context of sepsis, it does not completely rule out the existence of an underlying relationship between these two variables. Sepsis is a complex and multifaceted condition influenced by various factors, encompassing the infectious type, levels of inflammation, and the efficacy of early interventions. Depression may merely represent one contributing factor among a multitude of variables that influence the development of sepsis, while its impact on mortality rates might be overshadowed by other confounding factors. It is plausible that other latent mediators or moderators play significant roles within the relationship between depression and sepsis, thereby obscuring the observed association with mortality. This study utilized instrumental variables to probe the potential genetic causation between depression and sepsis. The findings highlight the substantial influence of depression on infection and the onset of sepsis, presenting optimistic implications for future healthcare. It is paramount to take into account the infection risk in individuals grappling with depression, underscoring the need for heightened vigilance in early patient assessments and monitoring protocols.

In future research endeavors, it is imperative to incorporate time-centric analyses. Exploring the prospect of an increased likelihood of infection either post-onset or preceding the manifestation of depression holds promise for valuable insights. Likewise, delving into correlations with the frequency of depressive episodes can illuminate nuanced aspects of the relationship. Furthermore, dissecting whether the risk of sepsis varies between isolated and recurrent episodes of depression promises to enrich our understanding. Encouragingly, future studies are urged to adopt more sophisticated indicators to capture the intricate temporal dynamics and potential nuances in the intricate interplay between depression and sepsis.

## Limitations

Our study has several limitations. Firstly, the GWAS data was sourced from a European population, raising questions about the applicability of your conclusions to non-European groups. Secondly, The mental health data gathered in registries are based on diagnoses made in psychiatric treatment settings, which may result in underreporting of depression. Additionally, the potential delay between the onset of depression and diagnosis may limit the accuracy of capturing this information in registry-based research. Thirdly, the inability to stratify the sample by sex, age, and region due to database constraints limits the exploration of your conclusion’s robustness across different subgroups. Fourthly, the link between depression and sepsis risk, as suggested by the MR method, is preliminary, and the underlying biological mechanisms remain unclear, necessitating further research for confirmation. Lastly, inconsistencies between causal results from methods like MR-egger and the main IVW method analysis suggest the presence of pleiotropy. Despite no clear evidence of horizontal pleiotropy in sensitivity analyses, verifying the hypothesis that genetic tools impact results solely through exposure factors, not vertical pleiotropy, is challenging. Moreover, a complete dismissal of pleiotropy is difficult due to the incomplete understanding of the biological effects of these SNPs.

## Conclusions

In conclusion, this study identifies depression as a causal risk factor for sepsis (not for sepsis-induced mortality), which means that individuals with depression need to pay more attention to the risk of sepsis.

## Supporting information

S1 Fig Leave-one-out sensitivity analysis. (A) Primary outcome (sepsis); (B) Secondary outcome (Sepsis mortality at 28 days).
(TIF)

S1 Table Instrumental variables. (XLSX)
