# Supplementary figures and images for: Identifying depression’s genetic role as a precursor to sepsis and increased mortality risk: Comprehensive insights from mendelian randomization analysis
Source: PLoS One. 2024 May 28;19(5):e0300275. doi: 10.1371/journal.pone.0300275 (PMC11132443; doi:10.1371/journal.pone.0300275)

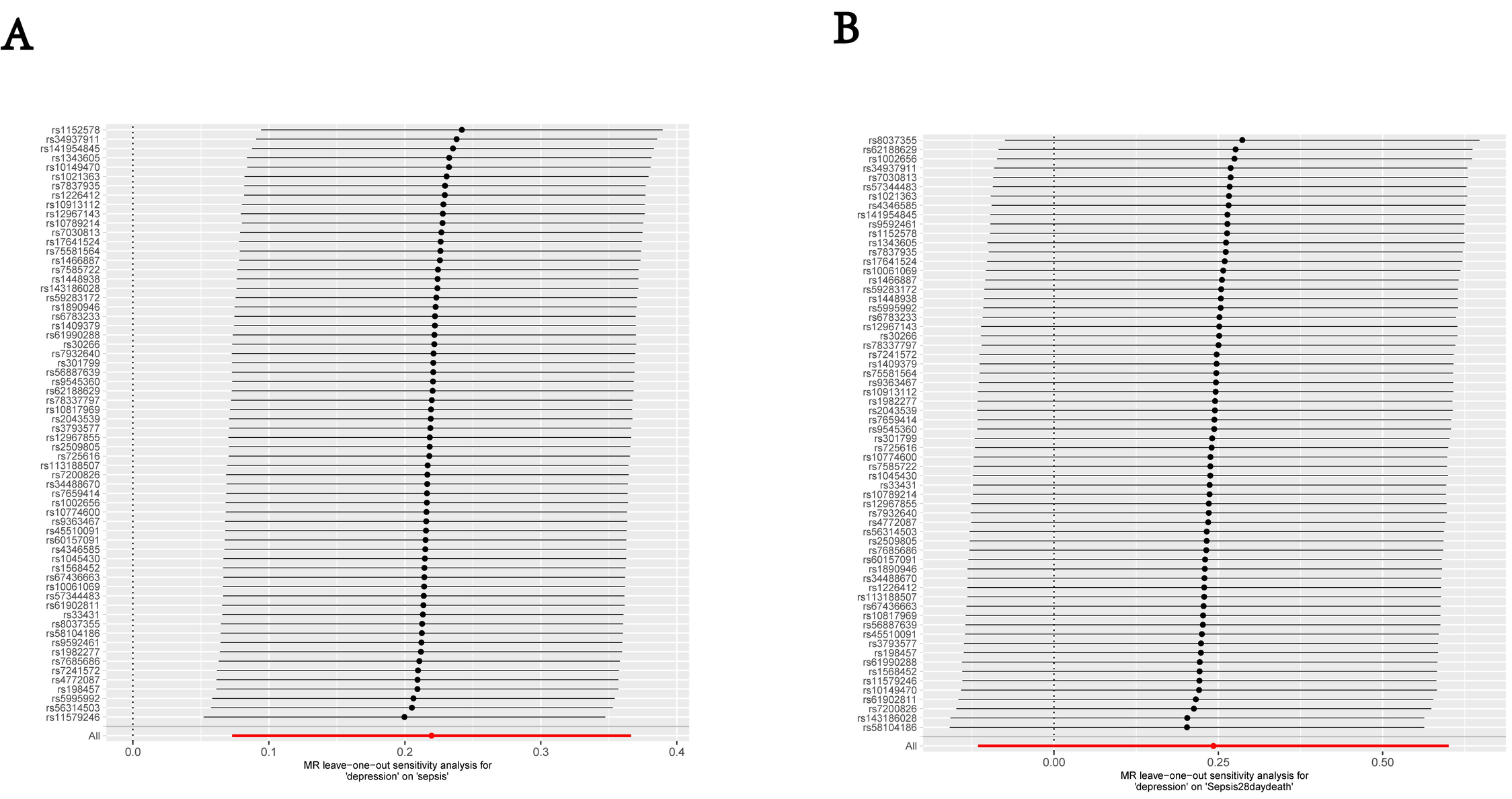

Supplement: S1 Fig — (A) Primary outcome (sepsis); (B) Secondary outcome (Sepsis mortality at 28 days). (TIF) [file pone.0300275.s001.tif]
